# ACEF score as a predictor of new-onset atrial fibrillation in patients with ST-elevation myocardial infarction: A retrospective cohort study

**DOI:** 10.1371/journal.pone.0339483

**Published:** 2025-12-19

**Authors:** Evliya Akdeniz, Burak Hünük, Cennet Yıldız, Süleyman Deniz Abdullahoğlu, Merve Ortakaya, Dilay Karabulut, Fatma Nihan Turhan Çağlar, Ömer Kozan

**Affiliations:** 1 Department of Cardiology, Bakirkoy Dr. Sadi Konuk Training and Research Hospital, Istanbul, Türkiye; 2 Department of Cardiology, Baskent University Istanbul Application and Research Center, Istanbul, Türkiye; Showa University: Showa Daigaku, JAPAN

## Abstract

**Background:**

New-onset atrial fibrillation (NOAF) during acute ST-elevation myocardial infarction (STEMI) is common and linked to adverse outcomes. This study aimed to assess the predictive value of the age–creatinine–ejection fraction (ACEF) score for NOAF in STEMI patients.

**Methods:**

We retrospectively analyzed 951 STEMI patients who underwent primary percutaneous coronary intervention (PCI) at out tertiary center between 2020 and 2023. Patients with prior atrial fibrillation (AF), AF on admission, cardiogenic shock, heart failure, significant valvular disease, or failed PCI were excluded. Clinical, laboratory, and echocardiographic data were collected, and ACEF scores were calculated. NOAF was defined as any AF episode during hospitalization in patients without prior AF. The optimal ACEF cutoff was determined using receiver operating characteristic (ROC) curve analysis (Youden’s index), and multivariate logistic regression identified independent predictors of NOAF.

**Results:**

NOAF occurred in 63 patients (6.6%). These patients were older, more often female, and had lower systolic blood pressure, lower left ventricular ejection fraction, and higher C-reactive protein and creatinine levels (all p < 0.05). In-hospital major adverse cardiac events (MACE) and mortality were significantly higher in the NOAF group than patients in without NOAF group (22.2% vs. 6.2%, and 15.9% vs. 4.3%, respectively; p < 0.001). The ACEF score was significantly higher in patients with NOAF (1.73 ± 0.74 vs. 1.42 ± 0.64, p < 0.001). ROC analysis showed an area under curve (AUC) of 0.643 (95% CI: 0.571–0.715; p < 0.001). An ACEF score ≥1.32 predicted NOAF with 73% sensitivity and 55.6% specificity. In multivariate analysis, ACEF remained an independent predictor of NOAF (OR ~1.5, p = 0.02), but among its components, only age was independently associated with NOAF (OR ~1.03 per year, p = 0.01).

**Conclusions:**

The ACEF score modestly predicts NOAF in STEMI patients, but its predictive power only incremental to age alone. Further studies are warranted for this field.

## Introduction

Atrial fibrillation (AF) is the most common arrhythmia in the setting of acute myocardial infarction (MI), with reported incidences ranging from 6% to 22% in the acute phase of MI [1]. New-onset atrial fibrillation (NOAF) during MI is associated with adverse outcomes, including increased in-hospital and long-term mortality [2–4]. The occurrence of AF in the setting of MI can complicate patient management, leading to longer hospital stays and a higher risk of complications such as heart failure and stroke. Given these implications, identifying patients at risk of developing NOAF after ST-elevation MI (STEMI) is an important clinical aim.

Various clinical, laboratory, and echocardiographic factors have been investigated as predictors of NOAF in MI patients. Prior studies have reported associations between NOAF and factors such as elevated natriuretic peptides, high-sensitivity C-reactive protein (CRP), uric acid–albumin ratio, stress hyperglycemia, neutrophil-to-lymphocyte ratio, certain echocardiographic measurements (e.g., transmitral E wave to early diastolic strain rate), left atrial size, and the extent of coronary artery disease [5–10]. While many individual markers have shown some predictive value, an ideal risk stratification tool should be easily obtainable, cost-effective, and be able to integrate multiple risk factors into a single score for practical use in daily practice.

The age–creatinine–ejection fraction (ACEF) score is a simple risk score originally introduced for mortality risk assessment in elective cardiac surgery patients [11]. It is calculated as age (years) divided by left ventricular ejection fraction (LVEF, %), with an additional point if serum creatinine > 2.0 mg/dL. Owing to its simplicity, the ACEF score has been evaluated in other cardiovascular contexts. In acute coronary syndromes including MI, previous studies have demonstrated that the ACEF score can predict major adverse cardiovascular events (MACE) and mortality, both short- and long-term. For instance, ACEF has shown prognostic value for 1-year mortality in MI patients undergoing PCI, and higher ACEF scores have correlated with increased mortality and MACE rates in various cohorts of patients with ischemic heart disease [12–16]. However, the relationship between ACEF and the risk of arrhythmic complications such as NOAF in STEMI remains unclear and has not been well studied to date.

In this study, we aimed to determine whether the ACEF score is associated with the development of NOAF in patients with STEMI treated with primary PCI. We also sought to compare the predictive performance of ACEF with traditional clinical risk scores and individual risk factors, and to evaluate the clinical implications of identifying high-risk patients.

## Materials and methods

### Study population and design

This retrospective cohort study included consecutive patients who were admitted to our tertiary care center with a diagnosis of STEMI between January 2020 and December 2023. Data access dates were 11/10/2024 and 28/11/2024. The inclusion criteria were: age ≥ 18 years, confirmed STEMI undergoing primary percutaneous coronary intervention (p-PCI) as the reperfusion strategy. The exclusion criteria were: any history of AF or presence of AF on the initial admission electrocardiography (ECG), cardiogenic shock on admission, overt congestive heart failure (Killip class III–IV) prior to p-PCI, moderate to severe valvular heart disease, and failed p-PCI (e.g., unsuccessful revascularization of the infarct related artery). These criteria were chosen to obtain a relatively homogeneous population of successfully reperfused STEMI patients without pre-existing arrhythmias or other conditions that could independently predispose to AF. A total of 1,024 patients were screened; 73 patients were excluded due to the criteria above (primarily prior AF or unsuccessful PCI), leaving 951 patients in the final analysis.

This study was approved by the local institutional ethics committee (Decision number: 2024-11-12, Approval date: 07 October 2024), and was conducted in accordance with the principles of the Declaration of Helsinki. All data were fully anonymized before we accessed them, and no identifiable personal information was used at any stage of the analysis. Written informed consent was obtained from all study participants prior to data collection. No minors were included in the study.

### Data collection and definitions

Baseline demographic information and clinical data were collected from the hospital’s electronic medical records and the national health database. Laboratory measurements at the time of emergency department admission, including cardiac enzymes, complete blood counts, electrolytes, renal function tests, inflammatory markers and lipid profiles were recorded. Each patient underwent a comprehensive transthoracic echocardiographic assessment during hospitalization (typically within 24–48 hours of admission) in accordance with guideline-recommended protocols [17]. STEMI was defined as the acute onset chest pain or equivalent symptoms suggestive of myocardial ischemia, accompanied by new or presumed new ST-segment elevation on a standard 12-lead electrocardiogram. Specifically, ST-elevation was defined as ≥1 mm (0.1 mV) in two or more anatomically contiguous leads, measured at the J-point, in all leads other than V2–V3. For leads V2–V3, the threshold for ST-elevation was defined as ≥2 mm in men aged ≥40 years, ≥ 2.5 mm in men aged <40 years, and ≥1.5 mm in women regardless of age [18]. LVEF was calculated using the biplane Simpson method. The ACEF score was then calculated for every patient using the formula: ACEF = Age/ LVEF + 1 (if serum creatinine > 2.0 mg/dL) [11].

NOAF was defined as any episode of AF (irregular rhythm with absence of distinct P waves on ECG, lasting >30 seconds) that occurred after hospital admission for STEMI, in a patient with no prior history of AF. Continuous cardiac monitoring was applied to all patients during their intensive care unit stay and standard 12-lead ECGs were obtained at least daily and whenever patients reported arrhythmic symptoms. NOAF episodes were verified by ECG documentation in the medical records. We recorded the timing of the first NOAF episode during hospitalization and any acute management required (e.g., pharmacological or electrical cardioversion) by reviewing nurse telemetry logs and physician notes. In-hospital MACE was defined as the occurrence of any major adverse cardiac event during the index hospitalization, including reinfarction, stroke, cardiogenic shock, or death (cardiovascular or all-cause).

There were minimal missing data in the dataset (<2% for laboratory values, which were mainly due to hemolyzed samples). These were addressed by repeat testing or, if unavailable, by carrying forward the last known value (for routine labs) or excluding the variable from analysis if critical (this was not required for any key variable). All 951 patients had complete data for the variables used in primary analyses, including age, LVEF, creatinine, and outcomes.

### Statistical analysis

All statistical analyses were performed using SPSS 26.0 (IBM Corp., Armonk, NY, USA). The distribution of continuous variables was evaluated with the Kolmogorov–Smirnov test. Continuous variables are presented as mean ± standard deviation or median with interquartile range (IQR) as appropriate. Categorical variables are presented as counts and percentages. For comparisons between two groups, the independent samples t-test was used for normally distributed continuous variables, and the Mann–Whitney U test for non-normal distributions. Categorical variables were compared using the chi-square test or Fisher’s exact test, as appropriate.

A receiver operating characteristic (ROC) curve analysis was conducted to assess the ability of age, ACEF score and CHA₂DS₂-VA score to predict NOAF and to determine the optimal cutoff value. The area under the ROC curve (AUC) was calculated with 95% confidence intervals (CI). We selected the threshold score that maximized the Youden index (sensitivity + specificity – 1) as the optimal cutoff for ACEF to discriminate NOAF occurrence.

Calibration of the ACEF score for predicting NOAF was assessed using decile-based grouping. The predicted and observed event rates were compared across deciles, and a calibration plot was generated by plotting the observed event rate against the mean predicted probability. A 45° reference line representing perfect calibration was added. Calibration was further evaluated using the Hosmer–Lemeshow goodness-of-fit test. Adequate calibration was indicated by non-significant test results (p > 0.05).

Univariate logistic regression analysis was performed to identify factors associated with NOAF. Variables with a p-value of <0.25 in univariate analysis were considered for multivariate modeling. Three multivariate logistic regression models were constructed to evaluate independent predictors of NOAF. In Model A, to explore the contribution of individual components of the ACEF score, we included age, LVEF, and creatinine separately (instead of the composite ACEF) along with other significant variables. In Model B and C, the ACEF score and CHA₂DS₂-VA score (as a continuous variable) was included respectively, along with other significant univariate predictors (if not collinear). The results of logistic regression are reported as odds ratios (OR) with 95% confidence interval (CI) and corresponding p-values. A two-tailed p < 0.05 was considered statistically significant.

## Results

### Patient characteristics and incidence of NOAF

In a cohort of 951 STEMI patients, 6.6% (63 patients) developed NOAF during hospitalization, with 48 episodes (76.2%) occurring within 48 hours. NOAF patients were significantly older (65.8 vs. 58.9 years, p < 0.001), more likely to be female (33.3% vs. 20.4%, p = 0.021), had lower systolic blood pressure on admission (129 vs. 137 mmHg, p = 0.027), reduced LVEF (43.1% vs. 46.2%, p = 0.036), higher CRP (4.00 vs. 2.73 mg/dL, p = 0.02), and elevated serum creatinine (1.15 vs. 0.97 mg/dL, p = 0.031). There were no statistically significant differences between patients who developed NOAF and those who did not with respect to KILLIP class (p = 0.563), final TIMI 3 flow (p = 0.246), or the distribution of the infarct-related artery (p = 0.397). The ACEF score was significantly higher in the NOAF group (1.73 vs. 1.42, p < 0.001), reflecting worse baseline risk profiles. The CHA₂DS₂-VA score was 2.48 ± 1.51 in patients without NOAF and 3.23 ± 1.68 in the NOAF group, with a significant difference between the groups (p < 0.001), (Table 1).

**Table 1:** Clinical and demographic characteristics of the study population, stratified by the occurrence of new-onset atrial fibrillation (NOAF). Data are presented as mean ± SD, median (IQR), or n (%).

Characteristic	All patients (n = 951)	Without NOAF (n = 888)	With NOAF (n = 63)	p-value
Age (years)	59.4 ± 12.6	58.9 ± 12.4	65.8 ± 14.0	<0.001
Female sex, n (%)	202 (21.2%)	181 (20.4%)	21 (33.3%)	0.021
Hypertension, n (%)	427 (44.9%)	393 (45.3%)	34 (54.0%)	0.166
Diabetes mellitus, n (%)	257 (27.0%)	236 (26.6%)	21 (33.3%)	0.243
CVA, n (%)	46 (4.8%)	42 (4.7%)	4 (6.3%)	0.817
Current smoker, n (%)	485 (51.0%)	462 (52.0%)	23 (36.5%)	0.017
SBP (mmHg)	136.8 ± 29.3			0.027
KILLIP class				0.563
KILLIP 1	665 (69.9%)	623 (70.2%)	42 (66.7)	
KILLIP 2	286 (30.1%)	265 (29.8%)	21 (33.3)	
LVEF (%)				0.036
C-reactive protein (mg/dL)	2.82 ± 4.32	2.73 ± 4.24	4.00 ± 5.23	0.02
Creatinine (mg/dL)	0.99 ± 0.67	0.97 ± 0.64	1.15 ± 1.03	0.031
TIMI 3 flow	870 (91.5)	815 (91.8)	55 (87.3)	0.246
Infarct related artery				0.397
Left anterior descending	526 (55.3%)	496 (55.9%)	30 (47.6%)	
Left circumflex	203 (21.3%)	186 (20.9%)	17 (27%)	
Right coronary artery	222 (23.3%)	206 (23.2%)	16 (25.4%)	
ACEF score	1.44 ± 0.65	1.42 ± 0.64	1.73 ± 0.74	<0.001
CHA_2_DS_2_-VA score	2.53 ± 1.53	2.48 ± 1.51	3.23 ± 1.68	<0.001
In-hospital MACE, n (%)	69 (7.3%)	55 (6.2%)	14 (22.2%)	<0.001
In-hospital mortality, n (%)	48 (5.0%)	38 (4.3%)	10 (15.9%)	<0.001

ACEF: age, creatinine, ejection fraction score; CHA₂DS₂-VA: Congestive heart failure (1 point), Hypertension (1 point), Age ≥ 75 years (2 points), Diabetes mellitus (1 point), Prior stroke or transient ischemic attack (2 points), Vascular disease (1 point), Age 65–74 years (1 point); CVA: cerebrovascular accident; LVEF: left ventricular ejection fraction; MACE: major adverse cardiovascular events; SBP: systolic blood pressure.

### In-hospital outcomes

Patients with NOAF experienced worse in-hospital outcomes than those without NOAF. The in-hospital mortality rate in the NOAF group was 15.9% (10 of 63 patients) versus 4.3% (38 of 888) in the non-NOAF group (p < 0.001). Similarly, in-hospital MACE occurred in 22.2% (14 patients) of NOAF patients, compared to 6.2% (55 patients) of patients without NOAF (p < 0.001). No significant differences in timing to reperfusion or infarct-related artery distribution were observed between those who did and did not develop NOAF suggesting that the development of NOAF was more related to patient-specific risk factors than to treatment delays.

As shown in Table 2, patients with a high ACEF score (≥1.32) had significantly worse clinical profiles and outcomes compared to those with lower scores. They experienced longer hospital stays (median 5 vs. 3 days, p = 0.004), higher incidence of NOAF (11.3% vs. 2.6%, p < 0.001). High ACEF scores were also associated with older age, lower LVEF, more comorbidities, elevated inflammatory and cardiac biomarkers, and worse in-hospital outcomes, including higher mortality (10.2% vs. 0.6%) and MACE (12.4% vs. 2.8%, both p < 0.001). High ACEF patients not only had a higher incidence of NOAF but also experienced earlier onset, with 70% developing AF within 24 hours compared to 31% in the low-risk group (p = 0.02), and a shorter median time to first AF episode (24 vs. 48 hours; Table 3). These findings highlight the ACEF score’s value in predicting both the likelihood and timing of NOAF, as well as broader adverse outcomes after STEMI.

**Table 2:** Comparison of patient characteristics and outcomes according to ACEF risk group. The cohort is divided into low-risk (ACEF <1.32) and high-risk (ACEF ≥1.32) based on the optimal cutoff from ROC analysis. Data are presented as mean ± SD or n (%).

Characteristic	ACEF Low Risk (n = 509)	ACEF High Risk (n = 442)	p-value
Age (years)	52.2 ± 8.7	67.6 ± 11.4	<0.001
Female sex, n (%)	68 (13.4%)	134 (30.3%)	<0.001
Hypertension, n (%)	164 (32.2%)	263 (59.5%)	<0.001
Diabetes mellitus, n (%)	90 (17.7%)	160 (37.8%)	<0.001
CVA, n (%)	16 (3.1%)	30 (6.8%)	0.022
Current smoker, n (%)	345 (67.8%)	140 (31.7%)	<0.001
LVEF (%)	52.6 ± 7.4	38.5± 9.5	<0.001
KILLIP class			<0.001
KILLIP 1	399 (78.4%)	266 (60.2%)	
KILLIP 2	110 (21.6%)	176 (39.8%)	
CRP (mg/dL)	2.15 ± 3.47	3.57 ± 5.02	<0.001
Peak troponin-T (ng/mL)	1224 (median)	1978 (median)	<0.001^a^
HbA1c (%)	6.53 ± 1.68	6.90 ± 1.87	<0.001
NT-proBNP (pg/mL)	176 ± 299	835 ± 1962	<0.001
Hemoglobin (g/dL)	14.13 ± 1.74	13.05 ± 2.11	<0.001
Sodium (mmol/L)	137.1 ± 3.7	135.9 ± 3.8	<0.001
CHA_2_DS_2_-VA score	1.79 ± 1.06	3.37 ± 1.56	<0.001
In-hospital NOAF, n (%)	13 (2.6%)	50 (11.3%)	<0.001
In-hospital mortality, n (%)	3 (0.6%)	45 (10.2%)	<0.001
In-hospital MACE, n (%)	14 (2.8%)	55 (12.4%)	<0.001
Length of stay (days), median (IQR)	3 (2-10)	5 (3-14)	0.004

^a^p-value for troponin is based on non-parametric comparisons (Mann–Whitney U test) due to non-normal distribution (median value shown for reference).

ACEF: age, creatinine, ejection fraction score; CHA₂DS₂-VA: Congestive heart failure (1 point), Hypertension (1 point), Age ≥ 75 years (2 points), Diabetes mellitus (1 point), Prior stroke or transient ischemic attack (2 points), Vascular disease (1 point), Age 65–74 years (1 point); CRP: C-reactive protein; CVA: cerebrovascular accident; HbA1c: hemoglobin A1c; IQR: interquartile range; LVEF: left ventricular ejection fraction; MACE: major adverse cardiovascular events; NOAF: new-onset atrial fibrillation; NT-proBNP: N-terminal pro–B-type natriuretic peptide.

**Table 3:** Timing of first NOAF episode in patients who developed NOAF, stratified by ACEF risk group. High ACEF scores were associated with earlier onset of atrial fibrillation after admission.

NOAF Timing (among NOAF patients)	Low ACEF (n = 13)	High ACEF (n = 50)	p-value
NOAF onset ≤24 hours, n (%)	4 (30.8%)	35 (70.0%)	0.020
NOAF onset >24 hours, n (%)	9 (69.2%)	15 (30.0%)	
Median time to first AF (hours, IQR)	48 (24-72)	24 (12-48)	0.033^b^

^b^p-value for difference in median time calculated by non-parametric test. AF: atrial fibrillation; NOAF: new-onset atrial fibrillation; IQR: interquartile range.

### Univariate and multivariate predictors of NOAF

In the univariate analysis, several clinical and laboratory variables were evaluated for their association with NOAF during hospitalization. Variables with p < 0.25, including age, female sex, systolic blood pressure, LVEF, CRP, hemoglobin, creatinine, CHA₂DS₂-VA score, and ACEF score, were considered for further multivariate analysis. Although KILLIP class 1 and 2 did not reach statistical significance in the univariate analysis, as shown in Table 2, the proportion of patients presenting with KILLIP 2 in the high-risk ACEF group was significantly higher compared to the low-risk ACEF group. Specifically, CHA₂DS₂-VA score (OR 1.333, 95% CI 1.143–1.554, p < 0.001), and ACEF score (OR 1.720 per 1-point increase, 95% CI 1.271–2.327, p < 0.001) were significantly associated with NOAF (Table 4).

**Table 4:** Univariate logistic regression analysis for predictors of NOAF in STEMI patients.

Variable	OR	95% CI	p-value
Age (per 1 year)	1.043	1.022-1.064	<0.001
Female sex	1.953	1.128-3.381	0.017
Smoking (current)	1.253	0.646-2.431	0.504
DM	1.085	0.779-1.511	0.629
Systolic BP (per 1 mmHg)	0.990	0.980-0.999	0.039
LVEF (per 1%)	0.975	0.953-0.998	0.030
CRP	1.053	1.005-1.102	0.028
Hemoglobin	0.913	0.807-1.033	0.148
Creatinine	1.258	0.985-1.605	0.066
KILLIP class 1–2	1.175	0.683-2.024	0.560
CHA_2_DS_2_-VA score	1.333	1.143-1.554	<0.001
ACEF score (per 1)	1.720	1.271-2.327	<0.001

ACEF: age, creatinine, ejection fraction score; BP: blood pressure; CHA₂DS₂-VA: Congestive heart failure (1 point), Hypertension (1 point), Age ≥ 75 years (2 points), Diabetes mellitus (1 point), Prior stroke or transient ischemic attack (2 points), Vascular disease (1 point), Age 65–74 years (1 point); CI: confidence interval; CRP: C-reactive protein; DM: diabetes mellitus; IQR: interquartile range; LVEF: left ventricular ejection fraction; MACE: major adverse cardiovascular events; NOAF: new-onset atrial fibrillation; OR: odds ratio.

In the multivariate analysis (Table 5), three models were constructed to explore the independent predictors of NOAF. In Model A, which included individual clinical and laboratory parameters, increasing age emerged as a significant predictor of NOAF (OR 1.026, 95% CI 1.001–1.052, p = 0.040), while other variables, including female sex, LVEF, CRP, hemoglobin, creatinine, and systolic blood pressure, did not reach statistical significance. When composite risk scores were incorporated, both the ACEF score (Model B; OR 1.569, 95% CI 1.086–2.266, p = 0.016) and the CHA₂DS₂-VA score (Model C; OR 1.262, 95% CI 1.040–1.532, p = 0.019) were independently associated with NOAF. Notably, female sex and CRP demonstrated trends toward association but did not achieve significance. These results suggest that while individual clinical and laboratory variables may provide limited predictive value, clinical risk scores such as ACEF and CHA₂DS₂-VA capture the cumulative burden of risk factors and identify patients at heightened risk for NOAF.

**Table 5:** Multivariate logistic regression models for independent predictors of NOAF.

	MODEL A		MODEL B (ACEF score)		MODEL C (CHA₂DS₂-VA score)	
	OR (95% CI)	p-value	OR (95% CI)	p-value	OR (95% CI)	p-value
Age	1.026 (1.001-1.052)	0.040	–	–	–	–
Female sex	1.732 (0.887-3.383)	0.108	1.823 (0.957-3.510)	0.067	1.654 (0.843-3.248)	0.144
LVEF	0.989 (0.964-1.015)	0.398	–	–	0.993 (0.967-1.019)	0.588
CRP	1.035 (0.982-1.091)	0.196	1.037 (0.985-1.093)	0.163	1.037 (0.984-1.094)	0.177
Hemoglobin	1.121 (0.952-1.320)	0.171	1.083 (0.927-1.264)	0.314	1.124 (0.955-1.322)	0.161
Creatinine	1.168 (0.871-1.568)	0.300	–	–	1.174 (0.868-1.588)	0.297
Systolic BP	0.991 (0.981-1.000)	0.061	0.992 (0.983-1.002)	0.116	0.991 (0.981-1.001)	0.063
ACEF score	–	–	1.569 (1.086-2.266)	0.016	–	–
CHA_2_DS_2_-VA score	–	–	–	–	1.262 (1.040-1.532)	0.019

Abbreviations as Table 4.

**Fig 1:**
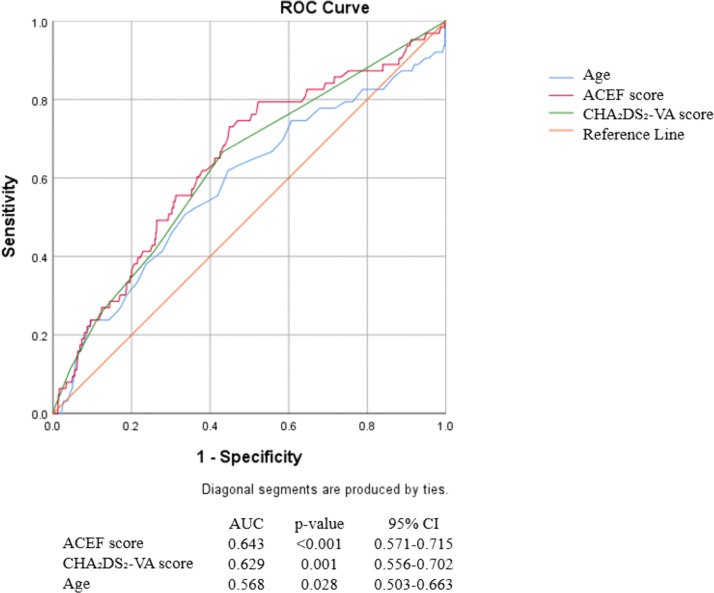
ROC curve analysis of age, ACEF score and CHA₂DS₂-VA score for prediction of NOAF in STEMI patients. The diagonal line represents the line of no-discrimination (AUC 0.50). ACEF: age, creatinine, ejection fraction; AUC: area under curve; CI: confidence interval; NOAF: new-onset atrial fibrillation; CHA₂DS₂-VA: congestive heart failure, hypertension, age ≥ 75 years, diabetes mellitus, prior stroke or transient ischemic attack, vascular disease, age 65–74 years.

The predictive performance of age, ACEF score and CHA₂DS₂-VA score for NOAF was evaluated by ROC curve analysis (Fig 1). The ACEF score showed a moderate ability to discriminate between patients who would develop NOAF and those who would not. AUC was 0.643 (95% CI 0.571–0.715, p < 0.001), indicating modest discrimination. The ROC analysis identified an optimal ACEF cutoff value of 1.32 for predicting NOAF in this population. This threshold was determined using the Youden index and represents the point at which the sum of sensitivity and specificity is maximized. An ACEF score ≥1.32 was considered “high risk” for the development of NOAF, whereas <1.32 was “low risk.” At this cutoff, the sensitivity for predicting NOAF was 73.0% and the specificity was 55.6%. The ACEF and CHA₂DS₂-VA scores both demonstrated significantly better predictive performance for NOAF than age alone (p = 0.011 and p = 0.035, respectively). There was no significant difference in discrimination between ACEF and CHA₂DS₂-VA scores (p = 0.682), indicating comparable predictive ability (Table 6).

**Table 6:** Comparison of predictive performance between age, ACEF, and CHA₂DS₂-VA scores for NOAF.

AUC comparison	z value	p-value	95% CI
Age vs ACEF score	−2.5445	0.011	−0.158 to −0.004
Age vs CHA_2_DS_2_VA score	−2.1078	0.035	−0.137 to −0.015
ACEF score vs CHA_2_DS_2_VA score	0.4087	0.682	0.005 to 0.026

AUC: area under the curve; ACEF: age, creatinine, ejection fraction score; CHA₂DS₂-VA: congestive heart failure, hypertension, age ≥ 75 years, diabetes mellitus, prior stroke or transient ischemic attack, vascular disease, age 65–74 years; CI: Confidence interval.

**Fig 2:**
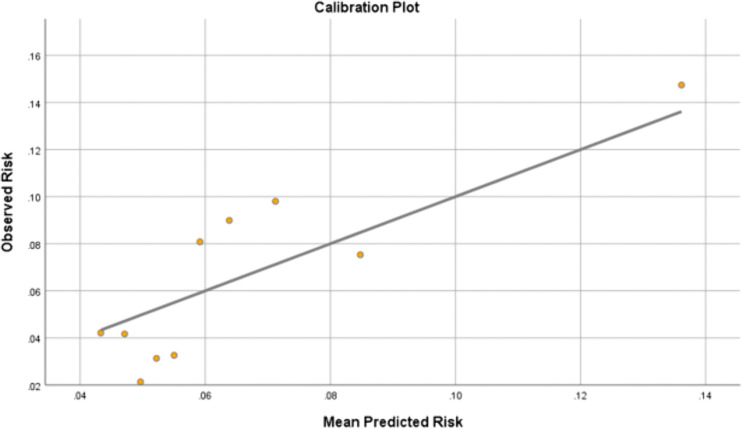
Calibration plot of the ACEF score.

The predicted and observed event rates showed a generally increasing trend across deciles, indicating reasonable agreement. A scatter plot was constructed with the observed event rate on the x-axis and the mean predicted probability on the y-axis. A 45° reference line representing perfect calibration was added, together with a linear regression line fitted to the data points. The plot showed good alignment with the reference line in the lower and mid deciles, while some degree of underestimation was observed in the highest decile. Both the decile-based calibration table and the calibration plot visually confirmed the high degree of concordance. The Hosmer-Lemeshow goodness-of-fit test indicated no significant lack of fit (χ² = 7.12, df = 8, p = 0.524), suggesting an adequate calibration of the ACEF score for predicting NOAF (Fig 2 and Table 7).

**Table 7:** Calibration of the ACEF score across deciles of predicted risk.

Decile	Mean predicted risk	Observed risk
1	4.3%	4.2%
2	4.7%	4.2%
3	4.9%	2.1%
4	5.2%	3.1%
5	5.5%	3.3%
6	5.9%	8.1%
7	6.4%	9.0%
8	7.1%	9.8%
9	8.5%	7.5%
10	13.6%	14.7%

## Discussion

This study evaluated the utility of the ACEF risk score in predicting NOAF in patients with STEMI. The key finding is that a higher ACEF score (≥1.32) is significantly associated with both an increased incidence and earlier onset of NOAF during the acute phase. While the ACEF score demonstrated moderate predictive performance, multivariate analysis indicated that its prognostic value is primarily driven by patient age. The positive predictive value of an ACEF ≥ 1.32 for NOAF was relatively low (given the low incidence of NOAF, many high-ACEF patients did not develop AF), and the negative predictive value was high (most low-ACEF patients truly did not develop AF).

Moreover, in our study with NOAF had significantly worse in-hospital outcomes than those without NOAF. In-hospital mortality and MACE rates were higher in the NOAF group than he non-NOAF group. These findings highlight the substantial clinical impact of NOAF during acute myocardial infarction, consistent with previous reports linking AF to increased morbidity and mortality [2,19,20].

### ACEF score as a predictor of NOAF

To our knowledge, this is one of the first studies to specifically evaluate the ACEF score for predicting in-hospital AF in STEMI patients treated with primary PCI. We found that ACEF was an independent predictor of NOAF in multivariate analysis (Model B), supporting the concept that this simple score, which combines age, LVEF, and renal function, captures elements of risk pertinent to arrhythmia development. Our results align with the general understanding that older patients with more comorbidities are at higher risk for AF in the setting of acute MI. Notably, the ACEF score has been previously validated as a prognostic tool for mortality and adverse events in various cardiovascular populations [12–16,21]. Its association with NOAF extends its potential applicability, highlighting that patients with a high ACEF score constitute a high-risk group not only for ischemic complications and death, but also for arrhythmic events.

Despite this, the absolute predictive performance of ACEF for NOAF was modest. The AUC of ~0.64 in our study indicates only fair discrimination. In practical terms, this means the ACEF score alone would not be a highly accurate test to predict which individual patients will develop AF. Our chosen cutoff of 1.32 provided decent sensitivity (73%) but low specificity (56%), implying a reasonably high negative predictive value. This level of performance limits the ACEF score’s stand-alone clinical utility for predicting NOAF. It is instructive to consider why a lower LVEF or higher creatinine might be expected to predict NOAF, even if in our study they did not emerge independently. A low LVEF can be associated with larger infarcts and higher left ventricular filling pressures, which in turn cause atrial stretch – a substrate for AF. Renal dysfunction often coexists with cardiovascular disease and may reflect a systemic disease burden and inflammation that promote AF [22,23]. In univariate analysis, we did see that lower EF and higher CRP were associated with NOAF. Thus, while these factors contribute to risk, they may operate in overlapping pathways with age or other comorbidities.

### Mechanisms linking AF with acute MI

Our study reinforces known mechanisms and associations in the context of AF during MI. We observed that NOAF patients had more evidence of hemodynamic stress (lower blood pressure, lower EF) and inflammation (higher CRP). There is a well-established pathophysiological basis for these observations. Acute MI can trigger AF through multiple, possibly synergistic pathways. Mechanical stress and atrial pressure overload: an acute loss of myocardium and reduced EF lead to elevated left ventricular end-diastolic pressure, pulmonary congestion, and increased left atrial pressure. The stretched atrial myocardium becomes more susceptible to AF. Additionally, in some cases of inferior MI, ischemia can involve atrial branches of the right coronary artery, causing atrial ischemia that may directly disrupt atrial electrophysiology. Prior studies have documented that atrial ischemia or infarction, while less common than ventricular involvement, can precipitate AF during MI [24,25].

Another component is the neurohormonal and inflammatory response that accompanies MI. Elevated sympathetic drive and release of catecholamines can increase atrial ectopy and shorten atrial refractory periods, facilitating AF. Inflammatory cytokines released during the acute phase of MI can alter cardiac conduction properties and promote AF as well. We found that CRP was higher in the NOAF group, though it did not remain an independent predictor in multivariate analysis. This could be due to the timing or sensitivity of CRP measurements. Our study used standard CRP assays; a high-sensitivity CRP might have better captured the inflammatory differences. Nevertheless, our findings are consistent with the notion that a pro-inflammatory state is associated with AF onset in the setting of MI, as others have reported [26–29]. Some inflammatory markers and leukocyte counts were numerically higher in NOAF patients (e.g., neutrophils) in our cohort, though not significantly.

### Clinical implications and management of high-risk patients

Identifying patients at risk for NOAF has important clinical implications, as those who develop NOAF in this study had significantly higher rates of adverse events and prolonged hospital stays. Patients with a high ACEF score (≥1.32) had a 50% increased odds of developing NOAF compared to those with lower scores. Importantly, most NOAF episodes in the high-ACEF group occurred early, often within the first 24 hours. These findings support a stratified monitoring approach—ensuring intensive telemetry for high-risk patients during the first 48–72 hours, while considering earlier step-down for stable, low-risk patients. Although no specific prophylactic therapy exists to prevent AF post-MI aside from guideline-directed beta-blocker use, the ACEF score may guide decisions on enhanced surveillance rather than pharmacologic intervention.

Our findings also have implications for post-discharge planning. NOAF during MI might be a one-time event, or it could unmask a predisposition to future AF. We did not follow patients long-term for recurrence of AF after discharge, but this remains an important question. Atrial fibrillation occurring in the context of an acute stress (so-called “secondary AF”) may not recur once the acute issue is resolved, but evidence is mixed. Some studies suggest that even transient NOAF during an acute coronary syndrome increases the risk of future AF and stroke after discharge [3,29]. Therefore, patients who had NOAF, especially those with high ACEF scores, might benefit from closer follow-up for arrhythmia detection and consideration of anticoagulation to mitigate stroke risk if AF recurs or persists. However, indiscriminate anticoagulation of all NOAF cases in MI is controversial, especially if AF was brief; current practice is to assess stroke risk factors (e.g., CHA₂DS₂-VA score) and individualize decisions. Interestingly, risk scores primarily developed for stroke risk in AF, like CHA₂DS₂-VA, share many components with ACEF, and one recent study found that CHA₂DS₂-VASc can independently predict NOAF in STEMI patients. This further underlines the overlap between profiles that predict thromboembolic risk and arrhythmic risk [30].

For high-risk patients (e.g., those with elevated ACEF scores), extended in-hospital observation and consideration of outpatient rhythm monitoring may be warranted, especially if AF is suspected but unconfirmed. These patients had longer hospital stays in our cohort, often due to AF or related complications. In response to reviewer inquiries, we support a cautious management approach: high-ACEF patients or those who develop NOAF should receive prolonged monitoring and a slower transition from the ICU until rhythm stability is assured. In contrast, low-risk, stable patients may be suitable for earlier ICU discharge, allowing for more efficient resource allocation.

Predicting NOAF is clinically valuable primarily for enabling early detection and timely management to prevent complications. In this study, all NOAF cases were successfully treated with standard care, without any in-hospital strokes or severe arrhythmic events. Although AF itself did not necessitate invasive interventions, it often coincided with other complications like heart failure, highlighting its role in overall patient complexity and care needs.

### Novelty and context of study findings

Compared to prior works, our study’s novelty lies in being the first (to our knowledge) to evaluate ACEF specifically for in-hospital AF prediction in a STEMI cohort. Previous studies on ACEF in MI have mostly focused on mortality, MACE, or long-term outcomes [12–14,16]. A few studies have indirectly touched on AF; for example, one study found ACEF could predict recurrence of AF after catheter ablation in a non-MI population, with an AUC (~0.624) similar to what we observed for NOAF prediction. This cross-context similarity suggests that ACEF consistently has modest predictive value for arrhythmia across different scenarios – sufficient to be statistically significant but not strong enough to be a standalone clinical decision tool.

In summary, this study demonstrates that the ACEF score is moderately effective in predicting NOAF in STEMI patients, correlating with both the likelihood and timing of NOAF, as well as in-hospital outcomes and resource utilization. However, multivariable analysis reveals that much of the ACEF score’s predictive value is attributable to age, a strong independent risk factor for AF,  LVEF and renal function did not reach statistical significance. While the ACEF score offers some improvement over age alone, its incremental predictive benefit is limited.

### Limitations

This single-center retrospective study has limitations including potential limited generalizability and focus solely on in-hospital NOAF without tracking long-term AF recurrence. Unmeasured confounders, such as infarct size or location, may influence AF risk. The ACEF cutoff of 1.32 was determined from our data and may vary in other cohorts, affecting predictive accuracy. A further limitation is the absence of granular data on AF duration, which limits the assessment of its association with in-hospital outcomes, such as MACE or mortality. Although patients who developed NOAF were anticoagulated with low-molecular-weight heparin during hospitalization, the absence of data on long-term oral anticoagulation represents an additional limitation of the study. External validation in larger, multi-center studies is needed. Ultimately, clinical impact requires trials testing whether interventions guided by ACEF risk stratification improve patient outcomes.

## Conclusion

In conclusion, our study shows that the ACEF score is moderately associated with NOAF in STEMI patients, mainly due to age. A score ≥1.32 identifies older, higher-risk patients who develop AF early and have worse in-hospital outcomes. However, ACEF adds limited predictive value beyond traditional risk factors. While useful for quick risk screening, it should not be solely relied upon for AF prediction or prevention. Further research is needed to improve prediction by combining ACEF with other markers and to evaluate targeted monitoring or treatment strategies for high-risk patients to improve outcomes.
