# Correction: ACEF score as a predictor of new-onset atrial fibrillation in patients with ST-elevation myocardial infarction: A retrospective cohort study

**DOI:** 10.1371/journal.pone.0342078

**Published:** 2026-02-02

**Authors:** Evliya Akdeniz, Burak Hünük, Cennet Yıldız, Süleyman Deniz Abdullahoğlu, Merve Ortakaya, Dilay Karabulut, Fatma Nihan Turhan Çağlar, Ömer Kozan

There is an error in Table 1.

**Table 1:** Clinical and demographic characteristics of the study population, stratified by the occurrence of new-onset atrial fibrillation (NOAF). Data are presented as mean ± SD, median (IQR), or n (%).

Characteristic	All patients (n = 951)	Without NOAF (n = 888)	With NOAF (n = 63)	p-value
Age (years)	59.4 ± 12.6	58.9 ± 12.4	65.8 ± 14.0	<0.001
Female sex, n (%)	202 (21.2%)	181 (20.4%)	181 (20.4%)	0.021
Hypertension, n (%)	427 (44.9%)	393 (45.3%)	34 (54.0%)	0.166
Diabetes mellitus, n (%)	257 (27.0%)	236 (26.6%)	21 (33.3%)	0.243
CVA, n (%)	46 (4.8%)	42 (4.7%)	4 (6.3%)	0.817
Current smoker, n (%)	485 (51.0%)	462 (52.0%)	23 (36.5%)	0.017
SBP (mmHg)	136.8 ± 29.3	137.4 ± 28.5	129.1 ± 38.6	0.027
KILLIP class				0.563
KILLIP 1	665 (69.9%)	623 (70.2%)	42 (66.7)	
KILLIP 2	286 (30.1%)	265 (29.8%)	21 (33.3)	
LVEF (%)	46.02 ± 10.9	46.2 ± 10.9	43.1 ± 11.0	0.036
C-reactive protein (mg/dL)	2.82 ± 4.32	2.73 ± 4.24	4.00 ± 5.23	0.02
Creatinine (mg/dL)	0.99 ± 0.67	0.97 ± 0.64	1.15 ± 1.03	0.031
TIMI 3 flow	870 (91.5)	815 (91.8)	55 (87.3)	0.246
Infarct related artery				0.397
Left anterior descending	526 (55.3%)	496 (55.9%)	30 (47.6%)	
Left circumflex	203 (21.3%)	186 (20.9%)	17 (27%)	
Right coronary artery	222 (23.3%)	206 (23.2%)	16 (25.4%)	
ACEF score	1.44 ± 0.65	1.42 ± 0.64	1.73 ± 0.74	<0.001
CHA2DS2-VA score	2.53 ± 1.53	2.48 ± 1.51	3.23 ± 1.68	<0.001
In-hospital MACE, n (%)	69 (7.3%)	55 (6.2%)	14 (22.2%)	<0.001
In-hospital mortality, n (%)	48 (5.0%)	38 (4.3%)	10 (15.9%)	<0.001
